# Probiotics and Isoflavones as a Promising Therapeutic for Calcium Status and Bone Health: A Narrative Review

**DOI:** 10.3390/foods10112685

**Published:** 2021-11-03

**Authors:** Iskandar Azmy Harahap, Joanna Suliburska

**Affiliations:** Department of Human Nutrition and Dietetics, Faculty of Food Science and Nutrition, Poznan University of Life Sciences, 60-624 Poznan, Poland; joanna.suliburska@up.poznan.pl

**Keywords:** probiotics, isoflavones, calcium status, bone health, osteoporosis

## Abstract

Probiotics have potential clinical effects for treating and preventing osteoporosis. Meanwhile, isoflavones have attracted much attention due to their ability to prevent postmenopausal symptoms. Research has established that probiotics and isoflavones can regulate hormones, immune cells, and the gastrointestinal system, acting as links in the gut–bone axis. However, combining the effects of probiotics and isoflavones on calcium status and bone health is a more novel and a still-evolving research area. *Lactobacillus* and *Bifidobacterium* are the foremost strains that influence bone health to a significant extent. Among the isoflavones, daidzein, genistein, and the metabolites of genistein (such as equol) stimulate bone formation. It can be concluded that probiotics and isoflavones promote bone health by regulating calcium uptake, gut microbiota, and various metabolic pathways that are associated with osteoblast activity and bone formation. Nevertheless, further experiments of probiotics and isoflavones are still necessary to confirm the association between calcium bioavailability and bone health.

## 1. Introduction

Osteoporosis is progressively becoming a very grave issue worldwide. This condition affects numerous people of all races and genders, and its occurrence will expand with the population, most frequently in Caucasians, women, and older people [1]. The gradual loss of bone with ageing is a normal condition. However, it may be accelerated by internal and external variables such as menopause, severe health conditions, and life factors such as an inadequate diet, insufficient exercise, smoking, or intemperate alcohol consumption [2]. On the one hand, the interactions between dietary calcium intake and bone health seem to differ; no consistent relationships have been demonstrated between dietary calcium and bone strength parameters. Besides, there may not be a significant association between dietary calcium intake and bone mineral density (BMD) in those with sufficiently (or high) vitamin D levels [3]. A positive interaction effect on the skeleton has also been seen between dietary protein and dietary calcium in older subjects who met their requirements but not those with lower calcium intakes [4]. On the other hand, there is acknowledged evidence of the interaction between calcium and vitamin D [5].

At the same time, vegetarians and vegans may be at greater risk of lower BMD and fractures than omnivores because of deficiency in calcium, vitamin D, vitamin B12, protein, and *n*-3 (*ω*-3) fatty acids in their diets. All of these nutrients play essential roles in maintaining bone health [6]. Furthermore, the association between calcium absorption and lactose intake [7] has not been clearly defined, nor between calcium absorption and caffeine intake [8]. However, fiber intake (soluble corn fiber and soluble fiber dextrin) has been found to increase calcium absorption [9].

A few current recommendations for treating and preventing osteoporosis among postmenopausal women are estrogen therapy and pharmacological agents. Estrogen treatment is also valuable in maintaining or improving bone mineral density [10] but presents an increased risk of reproductive system cancers [11]. However, pharmacological agents such as bisphosphonates, calcitonin, and denosumab (a receptor activator of nuclear factor-κB ligand / RANKL inhibitor) are not prescribed for long-term use, frequently require regular infusion appointments, and carry their own risk of adverse drug responses [12]. Furthermore, a meta-analysis has shown that a daily intake of calcium and vitamin D could decrease the risk of a total fracture by 15% and hip fracture by 30% [13]. It thus appears that clinicians’ current treatment and management of osteoporosis by employing dietary supplements alone may not be sufficient to prevent menopausal bone loss entirely.

Considering the duration of the treatment that would be needed to maintain bone health before and after menopause, it would be of great clinical value to create viable treatments with negligible side effects that are appropriate for longer-term use. Recent studies have found that probiotics and isoflavones have a significant impact on calcium absorption and bone health. With recent direct and indirect evidence in mind, this review focuses on the combination of probiotics and isoflavones and their effect on the host’s calcium status and bone health. There have been no high-quality reviews on this topic since 2010, to the best of our knowledge. We thus considered it necessary to describe the current state of knowledge on this very significant topic.

### 1.1. Probiotics

Probiotics are microorganisms that deliver health benefits to the host when consumed in the appropriate amounts [14]. The health benefits they deliver include potential clinical effects that are useful in treating and preventing osteoporosis [15]. Furthermore, probiotics could be used to decrease postmenopausal bone loss by increasing gut epithelial stability, increasing the expression of tight junction proteins, or reducing antigen transfer and lowering the activation of intestinal immune cells [16]. *Lactobacillus acidophilus* appears to be a promising strain with beneficial effects in ovariectomy [17] and osteoarthritis [18]. This strain is capable of colonizing the human colon, has antimicrobial effects, and can be used to treat intestinal infections [19]. Additionally, *L. acidophilus* has therapeutic potential as an osteoprotective agent in enhancing bone health. *L. acidophilus* in ovariectomized mice was able to improve both the trabecular and cortical bone microarchitecture, which also increased the mineral density and heterogeneity of bones. This effect of *L. acidophilus* administration is due to its immunomodulatory effect on the host immune system. *L. acidophilus* skews the Treg-Th17 cell balance by inhibiting osteoclastogenic Th17 cells and promoting antiosteoclastogenic Treg cells in ovariectomized mice. The administration of *L. acidophilus* also suppresses the expression of osteoclastogenic factors (interleukin 6 / IL-6, interleukin 17 / IL-17, tumor necrosis factor-alfa / TNF-α, and RANKL) and increases the expression of antiosteoclastogenic factors (such as interleukin 10 / IL-10, interferon gamma / IFN-γ) [17]. Moreover, *Lactobacillus casei* and *L. acidophilus* showed both the highest serum calcium level and the highest bone marrow concentration. This result indicated that the elevation of alkaline phosphatase (ALP), calcium (Ca), and phosphorus (P) is directly related to bone loss [20]. Therefore, these studies have shown a significant effect on various biochemical parameters that are triggered in postmenopausal osteoporosis and consequently prevent bone loss.

### 1.2. Isoflavones

Estrogen deficiency is a leading cause of bone loss and osteoporosis in postmenopausal women [21]. The three major chemical types of phytoestrogen that have been identified are isoflavones, lignans, and coumestans. The primary isoflavones in aglycone form are genistein, daidzein, and glycitein. These isoflavones can be found in soybeans and are considered potential alternatives to hormone therapy [22].

Daidzein, a soybean isoflavone, is metabolized to equol by the gut microflora in the gastrointestinal tract [23]. Nevertheless, daidzein reduces serum testosterone (T) and androstenedione (AD) levels in steroid metabolism between equol producing and non-equol producing women [24]. Unlike in women, both soy-based food and isoflavones have no consequence of free testosterone levels in men [25]. Equol itself may be involved in the regulation of androgens of the adrenal cortex in women [24]. Besides, equol inhibits bone loss, apparently without affecting the reproductive organs, in ovariectomized mice [26]. An association has also been found between the status of equol production and gut microbiota [27].

### 1.3. Calcium Status and Bone Health

Dietary supplements have become clinicians’ and patients’ preferred treatment for preventing and managing bone disease. The entry point of Ca into the body in humans and mammals is the intestine. There are two pathways for Ca absorption: through the paracellular pathway and the transcellular pathway [28]. First, a non-saturable process depends on the electrical gradient between the lumen and intestinal mucosa, allowing calcium ions’ passive diffusion across the intestinal epithelium [29]. Second, a transcellular saturable multistep process is initiated by transient receptor potential vanilloid type 6 (TRPV6). Once calcium has entered the cell through the TRPV6 channel, calcium-buffering proteins bind to the calcium and transport it. Finally, calcium is transferred from the cell to the blood vessels through plasma membrane ATPase 1b (PMCA1b) that is located in the basolateral membrane [30]. Hormones, nutrients, and other factors regulate both of the pathways. The major regulating hormone is calcitriol [1,25(OH)2D3], which works via vitamin D receptor signaling [31].

### 1.4. Gut Microbiota and Bone Health

The microbiota benefits the host’s health by producing essential nutrients, digesting food components, and enhancing the maturation’s immune system. However, dysbiosis, an unhealthy imbalance in the microbiota community composition, is linked to various metabolic, inflammatory, and immunologic diseases [32]. The imbalance of the gut microbiome can cause the imbalance of osteogenesis and osteoclast reaction [33]. When dysbiosis occurs, the gut microbiome loses its protective capabilities, and the gut barrier is impaired. The host fails to effectively control the dissemination of the gut microbiome components into the tissues [34]. The gut microbiota is critical for developing the immune system since both the microbiota and the immune system can regulate bone health [35]. Recent studies have shown that the gut microbiota significantly influences bone health (Table 1).

Hormones, immune cells, and the gastrointestinal system can regulate the balance of bone resorption by osteoclasts and bone formation by osteoblasts. Specifically, the gastrointestinal system contributes to absorbing bone mineralization [40], and it produces endocrine factors that signal to bone cells, such as incretins [41] and serotonin [42]. Probiotics that modify the microbiota composition or function and promote intestinal health can also benefit bone health [43]. The gut microbiome is essential for the efficient maturation of the immune system and cytoprotection against exogenous insults. The gut microbiota produces metabolites that account for anatomically distant biological effects. Indole derivatives were among the first bacterial metabolites to be described as affecting intestinal immunity. In addition, insulin-like growth factor 1 (IGF-1), produced predominantly in the liver in response to food intake and regulated by microbes and microbial products, was the first metabolite that was identified as a link in the gut–bone axis [44]. The crosstalk between the growth hormone/insulin-like growth factor-1 (GH/IGF-1) axis and the gut microbiota correlates with marked changes in microbial abundance (both phylum and genus shifts), richness, evenness, maturity, and levels of metabolites such as short-chain fatty acids, branched-chain amino acids, ammonia, and neurotransmitters [45].

## 2. Methods

**Figure 1:**
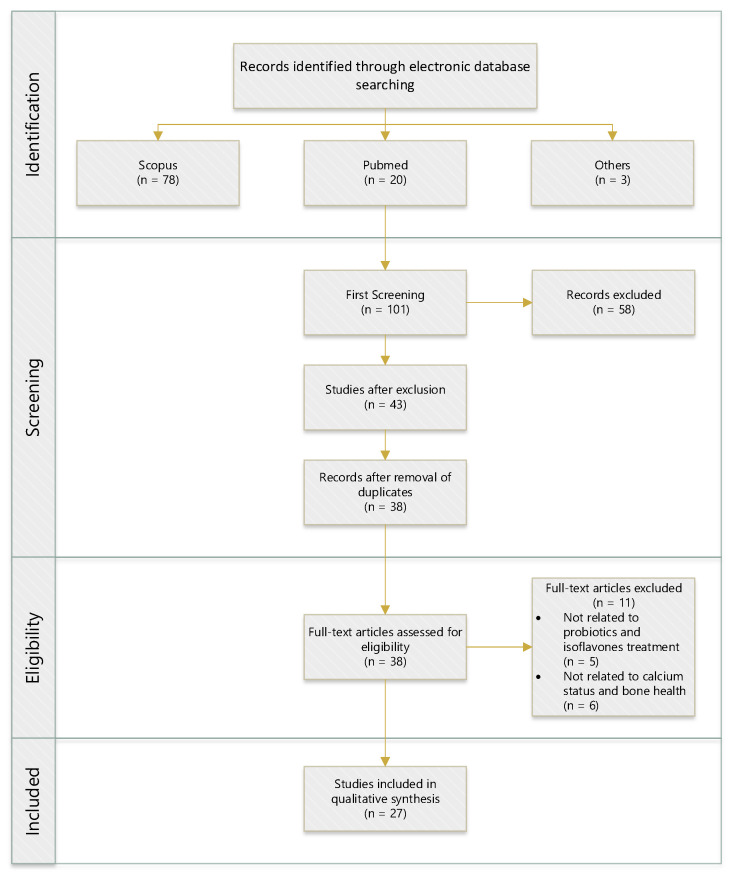
Flow diagram of the literature search and process of selection. (n = numbers).

The databases we searched included Medline via PubMed and Scopus. The search focused on citations after the year 2010 to capture the most recent evidence. To restrict the search results, we used the search terms (probiotic *) AND (isoflavone * OR daidzein OR equol) AND (calcium) AND (intake OR supplement * OR consum *) AND (bone OR osteo *) AND (cell * OR vitro *) AND (animal * OR rat * OR mice * OR mouse *) AND (human OR subject * OR volunteer * AND participant * OR women OR female). The inclusion and exclusion criteria were carefully chosen and are laid out in Table 2. The method encompassed in vitro, animal, and human studies in health and disease. Both observational and intervention studies were included. Studies with data duplication, lacking a description of their method, not in English, or published before 2010 were excluded, as were case reports. Therefore, this article was based on actual literature from the last ten years. Studies with other dietary supplements that were supplied simultaneously with probiotics or minerals were excluded. Figure 1 shows the search flowchart for the review.

### Data Synthesis

We synthesized the data narratively by summarizing the study design and mechanism findings in tables. The overall findings are explored in the Results (Section 3) and Discussions (Section 4).

## 3. Results

### 3.1. In Vitro Studies

Research on the effects of probiotics and their effects on the calcium status and bone health is limited compared to isoflavone in in vitro studies (Table 3). In vitro data are also helpful in determining the calcium status, bone health regulation, and other metabolites, elucidating their potential mechanisms. Probiotics and isoflavones may affect gastrointestinal health indicators, justifying animal and human studies.

Raveschot and colleagues [46] studied the probiotic properties of 174 *Lactobacillus* strains that were isolated from Mongolian dairy products and their impact on intestinal calcium uptake and absorption. A total of five *Lactobacillus* strains displayed good probiotic characteristics and modulated calcium absorption by intestinal cells—namely *L. casei* 9b, *L. kefiranofaciens* 15b, *L. plantarum* 46a, *L. helveticus* 49d, and *L. delbrueckii* 50b. *L. casei* 9b, *L. kefiranofaciens* 15b and *L. helveticus* 49d increased the total calcium transport by Caco-2 cells, most probably by improving calcium solubility. *L. delbrueckii* 50b impacted the paracellular pathway of calcium absorption by upregulation of the cld-2 gene. *L. plantarum* 46a improved the intestinal calcium uptake and absorption through the transcellular pathway involving VDR and TRPV6.

In another in vitro study on isoflavones, Fawwaz and colleagues [47] reported that soybean aglycone isoflavones were extracted by *L. bacillus* to test the effects on osteoblast cell proliferation in vitro, using a control, calcitonin, and natrium fluoride (NaF) as a positive control in this assay. The study results showed that NaF and calcitonin have 100% cell viability. The percentage of cell viability was taken to indicate the grade of cell proliferation; the greater the increase in osteoblast cell proliferation, the greater the increase in bone mass.

Daidzein shows promise as a potential antiosteoporosis agent. Hu and colleagues [48] determined the mechanisms underlying daidzein’s effects on osteoblast differentiation. The role of daidzein in bone morphogenetic protein (BMP)-2 gene expression was tested in organic cation transporter 1 (OCT1) cells. It was found to upregulate the expression of BMP-2, enhance the phosphorylated protein level of Smad1/5/8 and protein expression of Osterix (Osx, a direct target gene of BMP signaling), increase the activity of BMP signaling reporter (12xSBE-OC-Luc), and stimulate Col I, Runx2, and ALP expression. It could be concluded that daidzein acts by enabling the BMP-2/Smads pathway to promote osteoblast proliferation and differentiation.

Furthermore, daidzein stimulates osteogenesis through estrogen receptor-dependent signal pathways. An investigation was performed to compare the effects of daidzein and 17β-estradiol on the proliferation, differentiation, and cisplatin-induced apoptosis in human osteoblast-like MG-63 cells containing two estrogen-receptor (OR) isoforms. There were several effects of daidzein, including promoting cell viability, enhancing ALP activity and collagen type 1 content, and protecting against cisplatin-induced apoptosis in human osteoblast-like MG-63 cells [49]. Similarly, in human osteoblastic MG-63 cells, daidzein improved the protein and mRNA expression levels of osteoprotegerin (OPG) and simultaneously decreased the receptor activator of the nuclear factor-κB ligand (RANKL) and interleukin-6 (IL-6). Moreover, daidzein promoted the activation of the classic estrogen response element (ERE) pathway by increasing the expression of ERα, ERβ, and steroid hormone receptor coactivator (SRC)-1 [50]. Besides daidzein, Katsuyama and colleagues [51] reported a study that determined the beneficial effects of genistein or menaquinone-4 (MK-4) on osteoblastic MC3T3-E1 cell functions. They showed that GATA6, NOTCH2, and WNT5A were associated with osteoclast function. At the same time, the alterations in osteoblast function, BGLAP, and CHAD were increased in each treatment group at 48 h.

### 3.2. Animal Studies

Table 4 summarizes the experimental data that were collected from animal studies. Numerous animal studies have reported an altered calcium status and bone health following nourishment with probiotic and isoflavone ingredients. Parvaneh and colleagues [52] fed 24 10-week-old female mature Sprague-Dawley rats that were randomly grouped into a sham group, an ovariectomized group (OVX), and an OVX group that was supplemented with 1 mL of *B. longum* 10^8^–10^9^ CFU/mL. *B. longum* was given once daily for 16 weeks, starting from two weeks after the surgery. The effects of *B. longum* on bone mass density (BMD), bone mineral content (BMC), bone remodeling, bone structure, and gene expression in OVX rats were examined. The study concluded that *B. longum* increased the bone formation, decreased bone resorption, and altered the femur’s microstructure. The femur BMD was increased due to the upregulation of the Sparc and Bmp-2 genes.

Scholz-Ahrens and colleagues [53] tested whether the combination of a probiotic with a defined microbial strain resulted in improved bone mineralization and whether this effect was associated with gut ecology changes. A total of 80 ovariectomized adult rats were grouped into sham-operated group 1 and the ovariectomized groups 2–5. The rats were fed for 16 weeks on semi-purified diets containing 0.7% calcium and 0.5% phosphorus. Groups 1 and 2 received no supplements, group 3 was supplemented with a potential probiotic (*L. acidophilus* NCC90), group 4 was given prebiotics (oligofructose + acacia gum), and group 5 was given synbiotics (probiotics + prebiotics). The study demonstrated that the bone mineral loss following an ovariectomy was significantly prevented mainly by combining the specific prebiotic (oligofructose + acacia gum) with *L. acidophilus* NCC90.

Yang and colleagues [54] tested the beneficial effects of two novel *Lactobacilli* strain probiotics on bone health in ovariectomized induced osteoporotic mice and investigated its underlying mechanisms. A total of 45 9-week-old mice were grouped into a sham-operation (*n* = 9) or OVX (*n* = 36) groups. After four days post-operation, one group was treated with CMC (the control group), one was treated with alendronate at 2.5 mg/kg (the positive control), and the remaining two groups were orally treated with *Lactobacillus plantarum* GKM3 and *Lactobacillus paracasei* GKS6, both at a dose of 20.5 mg/kg. This study measured osteoporotic parameters by measuring the bone volume/tissue volume ratio, trabecular thickness, trabecular number, trabecular separation, and bone mineral density. The results showed that both of the probiotic strains inhibited bone loss, with GKS6 outperforming GKM3. Furthermore, GKS6 and GKM3 encouraged osteoblast differentiation via bone morphogenetic proteins (BMP) and inhibited RANKL-induced osteoclast differentiation through RANKL pathways. These findings demonstrated that *Lactobacilli* strains are potential candidates for treating and managing osteoporosis, particularly in postmenopausal osteoporosis.

Zhong and colleagues [55] tested the relationship between changes in intestinal flora and osteoporosis in rats with inflammatory bowel disease and the improvement effect of probiotics. A total of 100 Sprague-Dawley rats were randomly divided into two groups: a bowel disease group and an osteoporosis group (ovaries on both sides of the abdominal incision of all rats were removed), with 50 rats in each group. The osteoporosis group rats were randomly grouped into the control group (*n* = 25) and the experimental group (*n* = 25). The rats in the observation group underwent an enema with 12.5 g/kg *Lactobacillus rhamnosus*. The results showed that the serum values of OPG, PICP, TRACP, and Ca in the experimental group were higher than those in the control group. However, the serum values of RANKL, bone-specific alkaline phosphatase (BALP), IL-6, TNF-α, and INF-γ in the experimental group were lower than those in the control group. The authors concluded that the probiotics increased the value of serum inflammatory cytokines in rats; the activity of IL-6 and TNF-α may cause this with IL-6 stimulating the osteoclast precursors and TNF-α suppresses bone formation and osteoclast apoptosis. Whereas the occurrence of osteoporosis in rats with inflammatory bowel disease was positively related to the counts of *Lactobacillus* and *Bifidobacteria*. This study concluded that probiotics improved inflammatory bowel disease symptoms in rats with osteoporosis by influencing the level of the corresponding cytokines.

However, a study found that a combination of probiotics and polyphenol did not affect osteoporosis. Blanton [56] tested dietary enrichment with powdered whole grape and probiotics (composed of equal parts *Bifidobacterium bifidum*, *B. breve*, *Lactobacillus casei*, *L. plantarum*, and *L. bulgaricus*) on bone microarchitecture in a mouse model of age-related osteoporosis. Male mice that were ten months-old were grouped (*n* = 7 each) and fed one of six diets for six months: 10% grape powder with sugar corrected to 20%; 20% grape powder; 1% probiotic with sugar corrected to 20%; 10% grape powder + 1% probiotic with sugar corrected to 20%; 20% grape powder + 1% probiotic; and a 20% sugar control. The results showed that merging probiotics with 10% and 20% of grape diets exerted no effect on the bone microarchitecture measures, unlike independent probiotic and grape dietary enrichment. The authors concluded that there was no increased benefit to the bone by using the combined supplementation instead of the independent supplementation with probiotics or whole grape powder.

Unlike the above approaches, combining fermentation probiotics and the bioactive components of flavonoids showed a positive result in treating postmenopausal osteoporosis. Shim and colleagues [57] evaluated the effect of Hwangryun-haedok-tang (HRT) and its fermented product (fHRT), that was fermented by *Lactobacillus curvatus* KFRI-166, on postmenopausal bone loss using an ovariectomy rat model. The hormone replacement therapy contained 250 g *Coptis japonica Makino*, 250 g *Scutellaria baicalensis Georgi*, 250 g *Phellodendron chinense Schneider*, and 250 g *Gardenia jasminoides fructus*. Female Sprague-Dawley rats that were ten weeks-old were randomly grouped into sham-operated (sham, *n* = 8) and surgically ovariectomized (OVX, *n* = 24) groups. One week after surgery, the OVX rats were randomly assigned to an OVX that was administered with saline group 1; an OVX that was administered with 0.3 g/kg HRT group 2; and an OVX that was administered with 0.3 g/kg fHRT group 3. Inhibitory activity on RANKL-induced osteoclastogenesis by suppressing NFATc1 expression was found in the fHRT group, indicating an improvement in the BMD and bone parameters in the OVX rats. It was concluded that the administration of fHRT significantly slowed the decline of the bone mineral density and improved the femur bone parameters more than in the case of HRT and the bone parameter in OVX rats.

In another in vivo study on isoflavones, Tousen and colleagues [58] tested the combined effects of a diet that was supplemented with soy isoflavone (ISO) and resistant starch (RS) on intestinal microbiota, equol production, bone mineral density (BMD), and inflammatory gene expression in the bone marrow of ovariectomized (OVX) mice. Female ddY strain mice that were eight weeks-old were either sham-operated (*n* = 7) or underwent OVX on the same day. The OVX mice were placed into the following groups: (*n* = 7 each): OVX control (group 1); OVX fed 0.05% ISO-supplemented diet (group 2); OVX fed 9% RS-supplemented diet (group 3); and OVX fed 0.05% ISO-and 9% RS-supplemented diet (group 4). IL-7R and CD40L played a key role in bone resorption stimulated by estrogen deficiency. In the OVX mice, two weeks of diet supplementation with equol prevented an OVX-induced increase in IL-7R and CD40L. The authors concluded that ISO or its combination with RS supplement improved the bone marrow inflammation status, resulting in decreased bone loss in the OVX mice.

Furthermore, Tousen and colleagues [59] tested the combined effects of soy isoflavones (ISOs) and resveratrol (RES) on bone loss that was induced by hindlimb-unloading in mice. Female mice (ddY strain, 8 weeks) were randomly divided into six body weight-matched groups: a normally housed group (*n* = 6), a loading group (*n* = 6), a hindlimb-unloading group of mice that were fed a control diet (*n* = 6), aa hindlimb-unloading group of mice that were fed a 0.16% ISO conjugate diet (*n* = 8), a hindlimb-unloading group of mice that were fed a 0.15% RES diet (*n* = 8), and a hindlimb-unloading group of mice that were fed a 0.16% ISO conjugate and RES diet (*n* = 8). The ISO and RES treatment decreased the RANKL/OPG gene expression ratio in bone marrow cells in unloading mice. It also prevented the bone resorption that was caused by hindlimb-unloading by regulating RANKL and OPG mRNA expression in bone marrow cells.

Gautam and colleagues [60] investigated the effects of formononetin and cladrin (two structurally related methoxydaidzeins that are found in soy food and other natural sources) in osteoblast functions in bone formation in vivo. A total of 21 day-old immature female Sprague–Dawley rats were treated with 10.0 mg kg−1 body weight doses of an individual compound or vehicle (gum acacia in distilled water) once daily for 30 consecutive days by oral gavage. Each animal received an intraperitoneal injection of fluorochrome tetracycline (20 mg/kg body weight dose) and calcein (20 mg kg−1 body weight dose) on days 15 and 28 of treatment. This study analyzed the osteoblast proliferation, differentiation, and mineralization of bone marrow osteoprogenitor cells. The findings demonstrated that rats that were treated with cladrin had increased bone formation rates with the cladrin treatment but not in the control. Cladrin had much better plasma bioavailability than formononetin.

Tousen and colleagues [61] examined the effects of orally administered daidzein or equol on bone formation and bone mineral density in growing female rats. Female Sprague-Dawley rats, aged three weeks, were given 0.2mL of corn oil (the control group), 8 mg/day of daidzein, 4 mg/day of equol, or 8 mg/day of equol in a corn oil suspension (*n* = 8 per group). The results showed that daidzein and equol improved BMD in growing female rats by stimulating bone formation without showing a substantial effect on the weight of the reproductive organs. The bone growth was caused by increasing the mineralizing surface/bone surface ratio, and the bone formation rate in the equol group was approximately twice that of the rates that were observed in the daidzein group rats.

Abdelrazek and colleagues [62] tested the effects of soy isoflavones as hormone replacement therapy (HRT) on immunological and bone health. A total of 30 healthy cyclic female Wistar rats were grouped into a sham-operated group (*n* = 10) and an ovariectomy group (*n* = 20). The ovariectomized (OVX) female rats were randomly grouped into a control group that were fed a casein-based diet and the second group that were fed a high soy isoflavone diet (genistein and daidzein). Both of the groups were compared to a sham-operated group. Ovariectomy accelerated bone turnover, as manifested by increased ionized Ca^2+^ and phosphorous levels; decreased alkaline phosphatase activity would denote decrease osteoblast activity. Reduced calcitonin hormone levels also accompanied these changes. Elevated ALP activity may indicate active bone formation, as it is a byproduct of osteoblast activity. Hence, this study concluded that supplementing with soy isoflavones improved bone mineralization via the calcitonin hormone and improved lipid profile, and subsequently, the antioxidant reserve exerted an anti-inflammatory effect.

Lee and colleagues [63] investigated the effects of ovariectomy on the nutrikinetics of genistein metabolites. After eight weeks, female sham-operated and OVX mice (nine weeks old) were administered genistein (5 mg/kg body weight in 200 μL polyethylene glycol) via gavage. They found that the gut microbiota plays a significant role in the metabolism, bioavailability, and bioactivity of dietary compounds. The gut microbiota converts daidzein and genistein to equol.

### 3.3. Human Studies

Table 5 summarizes the experimental data that was collected from human studies. The results of several studies suggest that probiotics and isoflavones may have favorable effects on the calcium status and bone health for the treatment and prevention of osteoporosis in postmenopausal women. The study of Jafarnejad and colleagues [64] tested the effects of a multispecies probiotic supplementation on bone biomarkers and bone density in osteopenic postmenopausal women. This randomized, double-blind, placebo-controlled, clinical trial was performed on 50 patients with osteopenia aged 50–72. The participants were randomly grouped into a multispecies probiotic supplement group (GeriLact; *n* = 25) and a placebo group (*n* = 25) for six months. The GeriLact supplement contained seven probiotic bacteria species (*Lactobacillus casei, Bifidobacterium longum, Lactobacillus acidophilus, Lactobacillus rhamnosus, Lactobacillus bulgaricus, Bifidobacterium breve, and Streptococcus thermophilus*). The participants received 500 mg Ca plus 200 IU vitamin D daily. Decreases in BALP and collagen type-1 cross-linked C-telopeptide (CTX) levels and in serum parathyroid hormone (PTH) and tumor necrosis factor (TNF)-α were found in the intervention group but not in the placebo group.

Jansson and colleagues [65] investigated how the combination of three bacterial strains protects against rapid spine bone loss in healthy early postmenopausal women. A total of 249 participants were randomly assigned in a 1:1 ratio to receive probiotic treatment consisting of three Lactobacillus strains (*Lactobacillus paracasei* DSM 13434, *Lactobacillus plantarum* DSM 15312, and *Lactobacillus plantarum* DSM 15313; 1 × 10^1^⁰ CFU/capsule) or placebo once daily for 12 months. This study revealed that the loss of lumbar spine bone mineral density decreased in the *Lactobacillus*-treated group. It can be concluded that the probiotic treatment using a mix of three *Lactobacillus* strains protects against lumbar spine bone loss in healthy postmenopausal women.

Nilsson and colleagues [66] examined the effects of daily supplementation with *Lactobacillus reuteri* 6475 in bone loss in older women with low bone mineral density (BMD). A total of 90 subjects were enrolled and randomized to orally receive 10^10^ CFU of *L. reuteri* 6475 daily or placebo. In this randomized, placebo-controlled, double-blind, clinical trial, supplementation with *L. reuteri* 6475 for 12 months resulted in reduced bone loss in older women with low bone density. The experiment found that the daily supplementation with *L. reuteri* 6475 for 12 months reduced tibia total volumetric bone mineral density (vBMD) in older women with low BMD. It demonstrated that supplementation intake of *Lactobacillus* strain reserved bone mineral density in older women.

Takimoto and colleagues [67] examined the effect of the probiotic *Bacillus subtilis* C-3102 on BMD and gut microbiota in healthy postmenopausal Japanese women. A total of 76 participants were assigned an individual trial identification number and randomly allocated into two groups that were treated with placebo or C-3102 spore-containing tablets for 24 weeks. The experiment results showed that total hip BMD was enhanced in the C-3102 group compared with the placebo group. *Bifidobacterium* also increased in the C-3102 group compared with the baseline, and *Fusobacterium* decreased in the C-3102 group compared with the baseline. This study illustrated that probiotics improved BMD and modulated host-gut microbiota.

In another clinical trial with isoflavones, Lambert and colleagues [68] tested the beneficial effects of a bioavailable isoflavone and probiotic lactic acid bacteria treatment against postmenopausal osteopenia. This study was a 12-month parallel-design, placebo-controlled, double-blind, randomized controlled trial. A total of 78 postmenopausal osteopenic women that were supplemented with calcium (1200 mg/d), magnesium (550 mg/d), and calcitriol (25 mg/d) were given either red clover extract (RCE) (60 mg isoflavone aglycones/d and probiotics) or a masked placebo (control). The results demonstrated that estrogen deficiency attenuated BMD loss, improved bone turnover, and promoted a favorable estrogen metabolite profile (2-OH:16a-OH). Potent stimulation of equol was found in these women that were taking RCE twice-daily over one year.

Nayeem and colleagues [69] investigated how soy isoflavones affect bone mineral density (BMD). A total of 99 healthy premenopausal women were randomized to isoflavones (136.6 mg aglycone equivalence) and 98 to placebo for five days per week for up to two years. BMD and serum calcium of daidzein and genistein were measured before and during treatment. The result showed that daidzein had a similar but marginal effect, and genistein significantly decreased whole-body BMD at low normal serum calcium levels but increased whole-body BMD at higher serum calcium levels. This study showed that there was an interaction between the isoflavones and serum calcium on whole-body BMD changes.

Shedd-Wise and colleagues [70] examined the three-year effects of soy isoflavones on BMD and strength in postmenopausal women. A double-blind, randomized controlled trial in 224 eligible women examined the effects of two soy isoflavone doses (80 or 120 mg/d) or placebo tablets on vBMD and strength (using peripheral quantitative computed tomography) in healthy postmenopausal women (age 46–63). The study found that isoflavone supplements’ beneficial effects on the percentage change in the midshaft femur vBMD and on the midshaft femur strength-strain index improved for three years.

Zhang and colleagues [71] tested the effect of soy isoflavones that were combined with calcium on bone mineral density in perimenopausal Chinese women. A total of 160 perimenopausal women with osteoporosis or osteopenia were enrolled and randomized into four groups receiving control, soy isoflavone, calcium, or soy isoflavone that was combined with calcium. The isoflavone that was combined with calcium was found to decrease osteocalcin, luteinizing hormone (LH), follicle stimulating hormone (FSH), and malondialdehyde (MDA) levels, while increasing glutathione peroxidase (GSH) activity and serum calcium and vitamin D levels, as compared with the control, the isoflavone, and calcium groups. The authors concluded that isoflavone that was combined with calcium was effective and safe in attenuating BMD loss in perimenopausal women was better than soy isoflavone and calcium alone.

Kruger and colleagues [72] investigated the effects of green kiwifruit combined with isoflavones on equol production, bone turnover, and gut microflora in healthy postmenopausal women. Healthy women one to ten years after menopause were randomly allocated to group A (*n* = 16) or B (*n* = 17) for a 16-week crossover trial. A two-week lead-in period initiated the diet and was followed by two six-week interventions, with a two-week washout in between. Group A was fed isoflavones for the first six weeks, followed by isoflavones and kiwifruit for the following six weeks. In comparison, group B had the same intervention sequence in reverse. The participants received 50 mg isoflavones daily from an oral supplement containing daidzein and genistein. The results showed that kiwifruit supplementation did not alter the gut microbiota profile (*Bifidobacterium*, *Lactobacillales*, *Bacteroides*, *Prevotella*, and *Clostridium*). The absence of an effect on gut microbiota may be due to the interventions being of short duration. Isoflavone supplementation alone significantly increased serum undercarboxylated osteocalcin (ucOC), whereas isoflavone supplementation with kiwifruit had a beneficial effect by significantly decreasing serum ucOC.

## 4. Discussions

**Figure 2:**
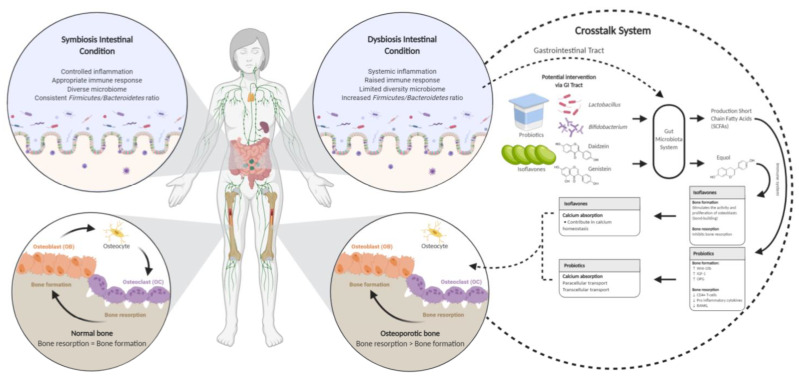
The crosstalk system mechanism of probiotics and isoflavones that benefits bone. An unhealthy balance in the microbiota community composition is linked to various metabolic, inflammatory, and immunologic diseases [32], and vice versa. The imbalance of the gut microbiome can cause the imbalance of osteogenesis and osteoclast reaction [33]. The gastrointestinal (GI) tract system contributes to absorbing bone mineralization [40]. The mechanism of action between probiotics and isoflavones regulates the GI tract system and calcium homeostasis. In the GI tract system, probiotics increase short-chain fatty acids [65] and elevate the immune system [55] to regulate the function of bone formation by increasing the level of Wnt Family Member 10B (Wnt10b), insulin-like growth factor 1 (IGF-1), and osteoprotegerin (OPG) [15] and lead the function of bone resorption by decreasing the level of CD4+ T cells, pro-inflammatory cytokines, and receptor activator of nuclear factor-κB ligand (RANKL) [15,54,55]. Furthermore, gut microbiota converts isoflavones (daidzein and genistein) to equol in the GI tract [63]. Isoflavones stimulate the activity and proliferation of osteoblasts [47] and inhibit bone resorption [26]. Isoflavones prevent bone resorption [58,59]. Isoflavones contribute to calcium homeostasis [70], and probiotic modulates the transcellular and paracellular pathways [46]. ↑: increase; ↓: decrease.

This literature review has examined a broad spectrum of evidence from studies of various designs to determine if probiotics and isoflavones affect calcium status and bone health. The demonstrated beneficial mechanism of probiotics and isoflavones in bone is shown in Figure 2. This figure illustrates the crosstalk mechanism of probiotics and isoflavones in the gut microbiota and calcium absorption.

In this review, the evidence on calcium status from in vitro studies suggests that the *L. plantarum* strain regulated the expression of vitamin D receptor and calcium transporter and modulated the transcellular pathway, while *L. delbrueckii* modulated claudin-2 expression on the paracellular pathway [47]. In human studies, micronutrients such as soy isoflavones represent a new class of compounds that can participate in calcium homeostasis by mobilizing calcium from bone into circulation [70]. Calcium absorption mostly occurs in the small intestine through paracellular and intracellular pathways. These calcium transport systems rely on the steroid hormone 1,25 dihydroxyvitamin D, and its interaction with the nuclear vitamin D receptor (VDR). The primary controlling hormone of intestinal Ca^2+^ transport is calcitriol [1,25(OH)2D3], which increases both pathways’ gene and protein expression [26,27,28,29].

Table 6 demonstrates the number of probiotics and isoflavones studies in previous experiments in vitro, animal, and human interventions to affect calcium status and bone health. The doses were spread out from 10^6^ CFU to 10^10^ CFU for probiotics and from 10 mg/kg to 4000 mg/kg for isoflavones. The results that were gathered in the current review established confirm the favorable impact of dietary consumption of probiotics and isoflavones on calcium absorption and bone health. It also showed that probiotics [73] and isoflavones [74] are safe to consume for humans, based on their safety profile according to general toxicity assessments.

Furthermore, in terms of bone health, the gut microbiota involves millions of bacteria and can be modified by numerous environmental factors, including diet. Numerous studies have established the bacteria in the human gut microbiota, including *Ruminococcaceae*, *Faecalibaculum*, *Lachnospiraceae*, and *Bacteroides*, as well as in bacterial phyla (e.g., *Actinobacteria*) and genera (e.g., *Lactobacillus* and *Bifidobacterium*) [45]. Ma and colleagues [69] reported that the identification of Firmicutes and Bacteroidetes is the gut microbiota’s main phylum. An increased *Firmicutes*/*Bacteroidetes* ratio after an ovariectomy can be identified as a useful biomarker for osteoporosis. Probiotics can alter the gut’s microbiota alignment and improve the solubility and absorption of minerals, leading to the immune system’s modulation. An example of a system that is modulated by probiotics is the process of bone re-modelling [53]. Rodent mechanistic experiments have investigated whether probiotics can reduce gut permeability, while increasing levels of short-chain fatty acids, reducing inflammation in the gut, reducing proinflammatory levels cytokines in bone, and reducing osteoclastic bone resorption [66]. Isoflavones are metabolized to equol by the gut microbiota in the intestinal tract [23]. Subsequently, equol increases bone mineral density by stimulating bone formation [69].

Overall, the evidence from human studies suggests that short-term or long-term probiotic and isoflavone treatment on bone health are minor compared with the effects of osteoporosis treatment with bisphosphonates [64,70] This evidence from in vitro, animal, and human studies generally suggests that probiotics and isoflavones can serve as a promising treatment or adjunct therapy for bone health issues or osteoporosis.

### Limitations

This study’s strength includes the updated scientific articles that considered probiotics and isoflavones supplementation within the last 10 years. It represents the beneficial effects of probiotics and isoflavones metabolism on calcium levels, bone health, and osteoporosis. We considered it essential to study the effects of probiotics and isoflavones because their dietary exposure is usually life-long. On the other hand, weaknesses of this review include pre-clinical data and observational studies in humans, such as risk factors, pathogenesis, and medications of bone health management, which would require a more comprehensive study. We did not explain the adverse event of probiotics and isoflavones as therapeutic management of bone health, whose effects would be anticipated to show the toxicity levels.

## 5. Conclusions and Future Trends

Both the in vitro and in vivo studies show that the probiotics that positively affect bone health are *Lactobacillus* and *Bifidobacterium*. The isoflavones that show most bone formation activity are daidzein and genistein, along with their metabolites, such as equol. In the calcium uptake, probiotics regulate calcium either on the transcellular pathway or on the paracellular pathway. Isoflavones participate in calcium homeostasis by mobilizing calcium from bone into circulation. In the gastrointestinal tract, *Lactobacillus* and *Bifidobacteria* improve the imbalance in the microbiota community composition and influence the immune system to regulate bone health. Isoflavones, including their metabolites, increase bone mineral density by stimulating bone formation.

From the authors’ viewpoint, future research and development could include several investigation areas that are particularly urgent, such as (1) developing nutritional and medical strategies to determine the prevention activity of probiotics and isoflavones as an epidemiological decision, (2) designing innovative products and diet recommendations of probiotics and isoflavones that correspond to the sensory characteristics, nutritional value, and physicochemical properties of older adults and postmenopausal women, and (3) exploring the natural resources of probiotics and isoflavones is crucial in designing novel dietary consumption to support the sustainable food systems. Finally, future research and industrial investments are vitally important to expand the application of probiotics and isoflavones to benefit food safety and public health.

## Figures and Tables

**Table 1:** Dysbiosis microbial signatures in bone health issues.

Study Design		Specific Dysbiosis Microbial Signatures	Ref
Human	48 primary osteoporosis 48 healthy	*Bacteroidetes* phylum, *Bacterioidia* class, *Bacteroidetes* order, *Ruminococcaceae* family, *Prevotellaceae* family, *Dialister* genus, and *Faecalibacterium* genus have been revealed as the key microbes related to primary osteoporosis	[33]
Human	6 Normal control (NC) (5F,1M) 6 Osteopenia (ON) (5F,1M) Osteoporosis (OP) (5F,1M)	Higher *Firmicutes* and lower *Bacteroidetes* were in the osteoporosis group than in the normal group. *Gemmatimonadetes* and *Chloroflexi* were different between bone health issues groups and control group	[36]
Human	108 postmenopausal women	*Klebsiella*, *Morganella*, *Escherichia/Shigella*, *Enterobacter*, *Citrobacter*, *Pseudomonas*, *Succinivibrio*, and *Desulfovibrio* were significantly higher in the postmenopausal osteopenia group	[37]
Animal	40 female Sprague Dawley	*Firmicutes/Bacteroidetes* Ratio, *Clostridium*, *Robinsoniella*, *Coprococcus,* and *Dialister* increased significantly after ovariectomy. *Ruminococcus flavefaciens* was the greatest abundance.	[38]
Animal	6 Male C57BL mice	A strong positive correlation was demonstrated between members of the *Actinobacteria phylum* (including the *Bifidobacteriaceae* family) and bone volume fraction ratio	[39]

**Table 2:** Study inclusion and exclusion criteria.

Parameter	Inclusion Criteria	Exclusion Criteria
Materials	In vitro, animal, and human studies, both in health and disease.	Not to be defined.
Intervention	Probiotic supply. Isoflavones supply.	Studies with other dietary supplements supplied simultaneously with probiotic, synbiotic, or isoflavones.
Comparator	Comparison of bacterial strains and isoflavones. Placebo or no comparator.	Not to be defined.
Outcomes	There are justifications for the use of probiotics and isoflavones, and they interact with the health of the host.	Not to be defined.
Study design	Intervention studies. Review articles only if they contain data on important issues not available elsewhere.	Case reports Studies duplicating data, lacking a description of method, not in English, or published before 2010.

**Table 3:** Summary of in vitro studies.

**Probiotic**	**Study Design**	**Mechanism Findings**	**Ref**
*L. casei, L. kefiranofaciens, L. plantarum, L. fermentum, L. helveticus and L. delbrueckii*	Six *Lactobacillus* strains from different species were selected, and their effect on intestinal calcium uptake and transport was investigated using Caco-2.	The *L. plantarum* strain modulates the transcellular pathway by regulating the expression of vitamin D receptor and calcium transporter. In contrast, the *L. delbrueckii* strain acts on the paracellular pathway by modulating claudin-2 expression.	[46]
**Isoflavones**	**Study Design**	**Mechanism Findings**	**Ref**
Genistein	Isoflavone aglycone was tested proliferation activity to osteoblast cell as in vitro	Genistein obtained as fermentation process of soybean by *Lactobacillus bulgaricus* is active to osteoblast cell proliferation.	[47]
Daidzein	The study examined the role of daidzein in the proliferation of OCT1 cells by the assay of XTT.	Daidzein enhanced the phosphorylated protein level of Smad1/5/8 and protein expression of Osterix (Osx, a direct target gene of BMP signaling) and increased BMP signaling activity reporter (12xSBE-OC-Luc).	[48]
Daidzein	The effects of daidzein compared with 17β-estradiol on proliferation, differentiation, and cisplatin-induced apoptosis in human osteoblast-like MG-63 cells containing 2 ER isoforms.	Daidzein promoted cell viability, enhanced ALP activity, collagen type 1 levels, and protected against cisplatin-induced apoptosis in human osteoblast-like MG-63 cells.	[49]
Daidzein	The study investigated the effects of daidzein, raloxifene, and E2 on expression of the osteoblast-produced bone regulatory factors OPG, RANKL, and IL-6 in human osteoblastic MG-63 cells.	Daidzein promoted the classic estrogen response element (ERE) pathway through increasing ERα, ERβ, and steroid hormone receptor coactivator (SRC)-1 expression.	[50]
Genistein and menaquinone-4 (MK-4)	The study evaluated the effects of genistein and MK-4 at obtainable dietary concentrations on the level of mRNAs and their protein products in MC3T3-E1 cells derived from neonatal mouse calvaria.	Genistein and/or MK-4 treatments increased BGLAP, indicating that this promoted an osteoblastic phenotype in the MC3T3-E1 cells.	[51]

**Table 4:** Summary of animal studies.

**Probiotic**	**Study Design**	**Mechanism Findings**	**Ref**
*Bifidobacterium longum*	The rats were randomly assigned into three groups (sham, OVX, and an OVX group supplemented with 1 mL of *B. longum* 10^8^–10^9^ CFU/mL). *B. longum* was given once daily for 16 weeks, starting two weeks after surgery.	Femur BMD increased due to the upregulation of Sparc and Bmp-2 genes.	[52]
*L. acidophilus* NCC90	80 ovariectomized adult rats were allocated to five groups: Group 1: sham-operated; groups 2–5: ovariectomized. Groups 1 and 2 got no supplements. Group 3 was given a potential probiotic (*L. acidophilus* NCC90), group 4 was fed prebiotics (oligofructose + acacia gum), and group 5 was fed synbiotics (probiotics + prebiotics).	Lowering pH has less impact on bone mineralization than the mass of digesta in the gut lumen and the mass of intestinal tissue. The luminal bowel content of the lower gastrointestinal tract is mainly composed of microbes known to release growth factors and to exert trophic effects on the intestine.	[53]
*Lactobacillus plantarum* GKM3, *Lactobacillus paracasei* GKS6	45 9-week-old mice underwent either a sham-operation (*n* = 9) or OVX (*n* = 36). In the four OVX groups, there were four groups (*n* = 9): the control group was treated with CMC; the positive control group was treated with alendronate at 2.5 mg/kg; the remaining two groups were orally treated with 20.5 mg/kg *L. plantarum* GKM3 and *L. paracasei* GKS6, respectively.	Both GKS6 and GKM3 promoted osteoblast differentiation and inhibited RANKL-induced osteoclast differentiation via bone morphogenetic proteins (BMP) and RANKL pathways, respectively.	[54]
*Lactobacillus rhamnosus*	Sprague–Dawley model rats with colitis were randomly divided into a control group (*n* = 25) and an observation group (*n* = 25). The observation group was treated with probiotics by gastric gavage, while the control group was treated with the same volume of physiological saline. The rats in the observation group underwent an enema with 12.5 g/kg *L. rhamnosus*.	*L. rhamnosus* elevates the level of serum inflammatory cytokines in rats to improve osteoporosis. IL-6 functions primarily in the early stage of osteoclast and stimulates the division and proliferation of osteoclast precursors. TNF-α is a bone absorption promoter, which suppresses bone formation and osteoclast apoptosis.	[55]
Powdered whole grape and probiotics (*Bifidobacterium bifidum, B. breve, Lactobacillus casei, L. plantarum,* and *L. bulgaricus*)	A group (*n* = 6) of mice was used to provide bones for baseline reference measurements to which age and diet-related changes in 16-month-old mice were compared. The remaining groups (*n* = 7) were fed one of six diets for six months (to age 16 months): 10% grape powder with sugar corrected to 20%; 20% grape powder; 1% probiotic with sugar corrected to 20%; 10% grape powder + 1% probiotic with sugar corrected to 20%; 20% grape powder + 1% probiotic; and 20% sugar control.	Dietary coenrichment with grape powder and probiotics does not produce a synergistic beneficial bone response in aging mice.	[56]
Hwangryun-haedok-tang (HRT), a Korean traditional herbal medicine fermented using *Lactobacillus curvatus*	Sprague-Dawley female rats (10 weeks old) were randomly divided into a sham-operated group (*n* = 8) and an OVX group (*n* = 24). The OVX rats were further assigned to three groups of eight rats each: (1) bilateral OVX administered with saline; (2) bilateral OVX administered 0.3 g/kg of HRT; (3) bilaterally OVX administered 0.3 g/kg of fHRT.	fHRT has inhibitory activity on RANKL-induced osteoclastogenesis by suppressing NFATc1 expression, resulting in an improvement of BMD and bone parameter in OVX rats.	[57]
**Isoflavones**	**Study Design**	**Mechanism Findings**	**Ref**
Soy isoflavone (ISO) daidzein with Resistant starch (RS)	Eight-week female ddY mice were randomly divided into five groups (*n* = 7 each): sham-operated; OVX control; OVX fed 0.05% ISO diet; OVX fed 9% RS diet; and OVX fed 0.05% ISO-and 9% RS diet. The supplemented ISO contained the purified ISO conjugates daidzin (55.8%), glycitin (27.3%), genistin (10.3%). and others (1.1%).	ISO and RS suppressed the increase in OVX-induced IL-7R mRNA expression and slightly decreased the expression of CD40L. IL-7R and CD40L play a crucial role in bone resorption stimulated by estrogen deficiency. ISO and combinations altered bone marrow inflammation status, resulting in attenuated bone loss in OVX mice.	[58]
Soy isoflavones (ISOs) and resveratrol (RES)	Eight-week female ddY mice were divided into six groups (*n* = 6–8 each): normally housed mice, loading mice, hindlimb-unloading (UL) mice fed a control diet, UL mice fed a 0.16% ISO conjugates, UL mice fed a 0.15% RES diet, and UL mice fed a 0.16% ISO and 0.15% RES diet.	ISO and RES prevent the bone resorption caused by hindlimb-unloading through regulating RANKL and OPG mRNA expression in bone marrow cells.	[59]
Cladrin and formononetin	Daily oral administration of each of these compounds at 10.0 mg/kg/day dose to recently weaned female Sprague-Dawley rats for 30 consecutive days increased bone mineral density at various anatomic positions studied.	Cladrin stimulated osteoblast proliferation and differentiation by activating the MEK-Erk pathway, while formononetin exerted its differentiation-promoting action by activating the p38 MAPK pathway.	[60]
Daidzein and equol	Female Sprague-Dawley rats, aged three weeks, were divided into four groups (*n* = 8 per group), orally administered corn oil, 8 mg/day of daidzein, 4 mg/day of equol, or 8 mg/day of equol in corn oil for four weeks.	Equol stimulates endocortical apposition as well as estradiol during the growth period.	[61]
Genistein and daidzein	30 healthy cyclic female Wistar rats were performed, where ten females were sham-operated, and twenty females were subjected to ovariectomy. The ovariectomized female rats were then randomly divided into two groups: the control group was fed a casein-based diet and the second was fed a high soy isoflavone diet. Both groups were compared to a sham-operated group fed a casein-based diet.	Ovariectomy accelerates bone turnover, which manifests as increased ionized Ca^2+^ and phosphorous levels, while decreased alkaline phosphatase activity denotes osteoblast activity. Elevated alkaline phosphatase activity may indicate active bone formation, as it is a byproduct of osteoblast activity.	[62]
Genistein	After eight weeks, the sham and OVX mice were administered genistein (5 mg/kg body weight in 200 μL polyethylene glycol) via gavage.	Gut microbiota converts daidzein and genistein to equol. Most of the circulating equol is in the form of glucuronidated or sulfated conjugates, which exert estrogen agonist activity.	[63]

**Table 5:** Summary of human studies.

**Probiotics**	**Study Design**	**Mechanism Findings**	**Ref**
7 bacteria species (*Lactobacillus casei*, *Bifidobacterium longum*, *Lactobacillus acidophilus*, *Lactobacillus rhamnosus*, *Lactobacillus bulgaricus*, *Bifidobacterium breve*, and *Streptococcus thermophilus)*	This randomized, double-blind placebo-controlled clinical trial was performed on 50 patients with osteopenia aged 50–72. Participants were randomly assigned to take either a multispecies probiotic supplement (GeriLact; n D 25) or placebo (*n* D 25) for six months.	Various strains of probiotics on bone may produce several short-chain fatty acids, which decrease PTH, followed by an increase in mineral absorption by solubilization. Probiotic administration reduces the expression of several proinflammatory and osteolytic cytokines (TNF-a and IL-1b).	[64]
*Lactobacillus paracasei* DSM 13434, *Lactobacillus plantarum* DSM 15312, and *Lactobacillus plantarum* DSM 15313	Early postmenopausal women were randomized to receive three *Lactobacillus* strains (1 × 10^1^⁰ CFU/capsule) or placebo once daily for 12 months.	The bone protective effect of probiotics reduces gut permeability, increases short-chain fatty acids, reduces inflammation in the gut, reduces levels of proinflammatory cytokines in bone, and decreases osteoclastic bone resorption.	[65]
*Lactobacillus reuteri* ATCCPTA 6475	In this double-blind, placebo-controlled study, women aged 75 to 80 with low BMD were randomized to orally receive 10^10^ colony-forming units of *L. reuteri* 6475 daily or placebo. The predefined primary end-point was a relative change after 12 months in tibia total volumetric BMD (vBMD).	*L. reuteri* 6475 for 12 months reduced loss of tibia total vBMD in older women with low BMD. The underlying mechanism for this has not been elucidated, and further studies are needed to evaluate this strain supplementation’s clinical usefulness.	[66]
*Bacillus subtilis* C-3102	76 healthy postmenopausal Japanese women were treated with a placebo or probiotic *B. subtilis* C-3102 spore-containing tablets for 24 weeks.	C-3102 improves BMD by inhibiting bone resorption and modulating gut microbiota in healthy postmenopausal women.	[67]
**Isoflavones**	**Study Design**	**Mechanism Findings**	**Ref**
Red clover extract (RCE) rich in isoflavone aglycones and probiotics (lactic acid bacteria)	A 12-month, double-blind, parallel design, placebo-controlled, randomized controlled trial of 78 postmenopausal osteopenic women supplemented with calcium (1200 mg/d), magnesium (550 mg/d), and calcitriol (25 mg/d) given either 60 mg isoflavone aglycones/d and probiotics (RCE) or a masked placebo (CON)	Twice-daily RCE intake over one year attenuated BMD loss caused by estrogen deficiency, improved bone turnover, promoted a favorable estrogen metabolite profile (2-OH:16a-OH), and stimulated equol production in postmenopausal women with osteopenia.	[68]
Daidzein, genistein, and glycitein	99 healthy premenopausal women were randomized to isoflavones (136.6 mg aglycone equivalence) and 98 to placebo for five days per week for up to two years. BMD, serum calcium and urinary excretion of daidzein and genistein were measured before and during treatment.	Isoflavone exposure interacted with serum calcium in affecting whole-body BMD, but not hip and spine BMD.	[69]
Genistein, daidzein, and glycitein	A double-blind, randomized controlled trial in healthy postmenopausal women (46–63 yr) were studied. There were two soy isoflavone doses (80 or 120 mg/d) vs. placebo tablets on volumetric bone mineral density and strength (using peripheral quantitative computed tomography)	Soy isoflavone exerted a modest beneficial effect on the percentage change in the midshaft femur vBMD as TLMP increased and a modest beneficial effect on the midshaft femur SSI as bone turnover (reflected by serum BAP) increased.	[70]
Soy isoflavone, calcium, and soy isoflavone combined with calcium	160 women with osteoporosis or osteopenia were enrolled and randomized into four groups, namely control, soy isoflavone, calcium, and soy isoflavone combined with calcium.	Isoflavone combined with calcium increases estradiol level and reduces osteocalcin level, while increasing plasma calcium concentration.	[71]
Daidzein and genistein and green kiwifruit	33 healthy postmenopausal Caucasian women were randomly allocated to two groups: Group A received isoflavones for the first six weeks, followed by isoflavones and kiwifruit for the following six weeks. Group B had the same intervention sequence in reverse. Isoflavone capsules and kiwifruit were taken in the morning with breakfast.	Osteocalcin (OC) is a vitamin K-dependent protein produced by the osteoblasts and is the primary noncollagenous protein in bone. Vitamin K acts as an essential cofactor for the enzymatic carboxylation of OC’s glutamyl side chains.	[72]

**Table 6:** Dosage intervention of probiotics and isoflavones in in vitro, animal, and human studies.

Study Design	Intervention	Dosage	Unit	Reference
Probiotics				
In vitro	*L. casei,* *L. kefiranofaciens,* *L. plantarum,* *L. fermentum,* *L. helveticus and* *L. delbrueckii*	10^7^	CFU/mL	[46]
Animal	*B. longum*	10^8^–10^9^	CFU/mL	[52]
	*L. acidophilus* NCC90	1–5 × 10^6^	CFU	[53]
	*L. plantarum* GKM3 and *L. paracasei* GKS6	2 × 10^11^	CFU/g	[54]
	*L. rhamnosus*	12.5	g/kg	[55]
	*B. bifidum*, *B. breve*, *L. casei*, *L. plantarum*, and *L. bulgaricus*	10^11^	CFU/g	[56]
	*L. curvatus* KFRI-166	0.3	g/kg	[57]
Human	*L. casei* 1.3 × 10^10^ CFU, *B. longum* 5 × 10^10^ CFU, *L. acidophilus* 1.5 × 10^10^ CFU, *L. rhamnosus* 3.5 × 10^9^ CFU, *L. bulgaricus* 2.5 × 10^8^ CFU, *B. breve* 1 × 10^10^ CFU, and *S. thermophilus* 1.5 × 10^8^ CFU	500	mg	[64]
	*L. paracasei* DSM 13434, *L. plantarum* DSM 15312, and *L. plantarum* DSM 15313	1 × 10^1^⁰	CFU	[65]
	*L. reuteri* ATCCPTA 6475	1 × 10^1^⁰	CFU	[66]
	*Bacillus subtilis* C-3102	3.4 × 10^9^	CFU	[67]
**Isoflavones**				
In vitro	Daidzein	0.001; 0.005; 0.01; 0.03; 0.06	mg	[48]
	Daidzein	0.01; 0.1; 1; 10	μmol/L	[49]
	Daidzein	0.01; 0.1; 1	μM	[50]
	Genistein	1	µM	[51]
Animal	Daidzin (55.8%); glycitin (27.3%); genistin (10.3%) and others (1.1%).	530	mg/kg	[58]
	Daidzein (33 mg); genistein (8.5 mg); and glycitein (15 mg)	4000	mg/kg	[59]
	Cladrin and formononetin	10	mg/kg	[60]
	Daidzein	8	mg	[61]
	Genistein; daidzein	1500; 800	mg/kg	[62]
	Genistein	5	mg/kg	[63]
Human	Red clover extract (RCE) rich in isoflavone aglycones and probiotics (lactic acid bacteria)	60	mg	[68]
	Daidzein; genistein; glycitein	30; 30; 8.3	mg	[69]
	Genistein:daidzein:glycitein (1.3:1:0.3)	80 and 120	mg	[70]
	Soy isoflavone; calcium	15; 125	mg	[71]
	Daidzein and genistein	50	mg	[72]

## Data Availability

The datasets generated for this study are available on request to the corresponding author.
